# Disentangling manual muscle testing and Applied Kinesiology: critique and reinterpretation of a literature review

**DOI:** 10.1186/1746-1340-15-11

**Published:** 2007-08-23

**Authors:** Mitchell Haas, Robert Cooperstein, David Peterson

**Affiliations:** 1 Western States Chiropractic College, 2900 NE 132^nd ^Avenue, Portland, OR 97230, USA; 2 Palmer College of Chiropractic West, San Jose, CA 95134, USA

## Abstract

Cuthbert and Goodheart recently published a narrative review on the reliability and validity of manual muscle testing (MMT) in the Journal. The authors should be recognized for their effort to synthesize this vast body of literature. However, the review contains critical errors in the search methods, inclusion criteria, quality assessment, validity definitions, study interpretation, literature synthesis, generalizability of study findings, and conclusion formulation that merit a reconsideration of the authors' findings. Most importantly, a misunderstanding of the review could easily arise because the authors did not distinguish the general use of muscle strength testing from the specific applications that distinguish the Applied Kinesiology (AK) chiropractic technique. The article makes the fundamental error of implying that the reliability and validity of manual muscle testing lends some degree of credibility to the unique diagnostic procedures of AK. The purpose of this commentary is to provide a critical appraisal of the review, suggest conclusions consistent with the literature both reviewed and omitted, and extricate conclusions that can be made about AK in particular from those that can be made about MMT. When AK is disentangled from standard orthopedic muscle testing, the few studies evaluating unique AK procedures either refute or cannot support the validity of AK procedures as diagnostic tests. The evidence to date does not support the use of MMT for the diagnosis of organic disease or pre/subclinical conditions.

## Background

Cuthbert and Goodheart recently published a narrative review on the reliability and validity of manual muscle testing (MMT) in the Journal [1]. They concluded that "The MMT employed by chiropractors, physical therapists, and neurologists was shown to be a clinically useful tool, but its ultimate scientific validation and application requires testing that employs sophisticated research models in the areas of neurophysiology, biomechanics, RCTs, and statistical analysis." The authors included Applied Kinesiology (AK) applications under the rubric of MMT.

The authors should be recognized for their effort to synthesize this vast body of literature. However, the review contains critical errors in the search methods, inclusion criteria, quality assessment, validity definitions, study interpretation, literature synthesis, generalizability of study findings, and conclusion formulation that merit a reconsideration of the authors' findings. Most importantly, a misunderstanding of the review could easily arise, because the authors did not distinguish the general use of manual muscle strength testing from the specific applications that distinguish the AK chiropractic technique. The purpose of this commentary is to provide a critical appraisal of the review to expose important flaws, suggest conclusions consistent with the literature reviewed and omitted, and disentangle conclusions that can be made about AK in particular from those that can be made about MMT. Note that we have not conducted a full systematic review.

## Discussion

### Appraisal elements

The validity of this review of MMT inevitably depends on the quality of the review process. It does not appear to have been the intent of the authors to conduct a full systematic review of the literature, and we do not hold them to that standard. However, design elements of a good systematic review of diagnostic tests [2-4], as well as critical appraisal of the measurement evaluation literature [5-14], are pertinent to the discussion at hand. Even the more traditional narrative review shares many of these elements [15]. We have compiled questions that must be considered in order to draw valid inference on the usefulness of AK diagnostic procedures (Table 1); these questions are based on research and synthesis methodology from the citations above. The answers to these few questions pose a serious challenge to the authors' conclusion about the usefulness of AK.

**Table 1:** Critical appraisal questions for Applied Kinesiology (AK)

**Literature review**
1. Were clear clinical questions identified and answered?
2. Were clear and appropriate inclusion/exclusion criteria identified and followed?
3. Was a thorough literature search conducted that retrieved all the pertinent literature?
4. Was an appropriate literature synthesis method identified and properly applied?
5. Was the literature interpreted properly?
6. Were the authors conclusions correct?

**Measurement evaluation (test usefulness)**
1. Was the reliability of AK diagnostic procedures determined to be adequate?
2. Was the construct validity of AK procedures identified?
3. Was the sensitivity and specificity or likelihood ratios of AK diagnostics for the identification of clinical procedures shown to be adequate?
4. Was the clinical utility of AK diagnostic procedures evaluated?
5. Was the efficacy of AK diagnostic procedures determined?

### AK entanglement

AK has a long and rich history in chiropractic [1,16]. Many chiropractors report use of the technique in some form [17,18]. Clearly, AK is viewed by its proponents as more than standard orthopedic/neurological muscle testing. MMT, as performed by chiropractors, does not necessarily differ in its execution and interpretation from manual muscle testing as performed and interpreted by the standards applied in physical medicine. To either practitioner, a weak muscle might suggest a primary muscular or neurological pathology. However, AK technique uses manual muscle testing not just to evaluate the functional integrity of muscle and nerve supply, but also as a means to "diagnose structural [and functional], chemical, and mental dysfunctions [1]." Some of its distinguishing diagnostic procedures include the use of provocative tests (i.e., AK challenge and therapy localization) in conjunction with MMT to identify the need for treatment of neuromusculoskeletal, organic, and metabolic conditions [19-21]. Muscle weakness is also considered diagnostic of pre/subclinical organic, non-neuromusculoskeletal disease.

MMT is a standard component of the neuromusculoskeletal physical examination [22]. We agree with the authors that MMT is useful in the assessment of weakness of muscles directly involved with pain, injury, and neuromusculoskeletal disorders. However, extrapolation of MMT properties to unique AK applications is risky for several reasons. MMT reliability/validity for specific neuromusculoskeletal conditions may not be generalizable to other applications such as identification of organic disorders. MMT may be reliable/accurate for muscle strength assessment in isolation, but not when used in conjunction with a spinal challenge (force applied to a vertebral articulation) or other provocative test used for specific AK diagnosis. The authors also confuse two uses of the term validity: test accuracy and diagnostic validity. A test may be extremely accurate, let us say for example dynamometric evaluation of muscle force in newtons, but still have no sensitivity or specificity for the diagnosis of a specific condition [5,6]. Cuthbert and Goodheart conflated evidence for AK with evidence of the reliability/validity of standard orthopedic MMT. The reliability and accuracy of MMT does not establish the usefulness of MMT for its unique AK applications.

### Search strategy and inclusion criteria

The review by Cuthbert and Goodheart illustrates how failure to utilize a fastidious search strategy can miss critical citations and impact review findings. The authors conducted an online search of PubMed and CINAHL, using the search terms "manual muscle test" and "manual muscle testing." No further details were provided, so the search cannot be duplicated exactly. There are several problems pertaining to the scope of the search that may have led to the omission of relevant articles. In our search of PubMed, the addition of the search term "muscle testing" increased the number of papers found from 639 to 13,802. We also conducted a search using MEDLINE and CINAHL. Including the additional term "muscle testing" increased the number of hits from 454 to 709, and the number of papers specifically pertaining to reliability/validity from 97 to 136. The second problem is that Cuthbert and Goodheart failed to search the chiropractic database, MANTIS. Including a search of this database increased the number of muscle testing papers from 709 to 1297 and the reliability/validity-related papers from 136 to 221. We also conducted a search using the Boolean strategy: Applied Kinesiology AND (reliability OR validity). The inclusion of MANTIS increased our yield from 15 to 32 articles. The authors may also have failed to use another important search strategy, namely checking article references to identify further pertinent studies.

The authors stated that they selected studies based on relevance, but did not include an operational definition. It appears that any MMT article on a pain-related disorder was considered relevant. It is not clear how "reliability/validity" and "MMT" were used in the selection process. Negative studies were certainly omitted. Had the authors used the search term "muscle testing" and included the MANTIS database, they would not have failed to identify randomized trials designed specifically to evaluate the contribution of an AK-challenge procedure to MMT results [23-25]. In any event, the authors should have been aware of the 1982 study by Triano that was conducted with the assistance of the International College of Applied Kinesiology [23] and critiqued by Goodheart in a letter to the editor [26].

One selection criterion introduced clear and significant bias into the review. Studies were only included if a kappa ≥ 0.5 was reported for the assessment of reliability or validity (though kappa is not generally a validity index). Clearly this inclusion criterion was not uniformly applied, since many of the included studies did not address reliability and thus did not report a kappa value. More importantly, the use of this criterion was based on a misunderstanding of Swinkles et al [27]. These authors used the criterion for setting standards for determining whether certain instruments had good construct validity; they did not use a threshold of kappa ≥ 0.5 to identify eligibility for their systematic review. The result of using this kappa selection criterion by Cuthbert and Goodheart was the exclusion of all but the studies with moderate to excellent reliability/validity. The biased inclusion criterion clearly set up a tautology that pre-determined a positive conclusion about the usefulness of MMT.

### Quality evaluation and evidence synthesis

Evaluation of study quality is an important aspect of literature reviews [15,28], and certainly there are many methods for doing this [29]. Cuthbert and Goodheart write in the methods section that a quality assessment was performed. It is not until the end of the paper that the authors acknowledge that internal and external validity have not been critically evaluated. The authors had no formal criteria or algorithm for synthesizing the literature to reach a conclusion about MMT in general and AK specifically. Without quality assessment, studies of great merit are inevitably given no more weight than studies with serious design flaws and unsupported conclusions. In particular, it is not advisable to take authors' conclusions from included articles at face value. Misinterpretations occur. Some examples in the chiropractic literature of conclusions inconsistent with study design and results are identified in several reviews [9,30,31].

### Evidence from treatment investigations

Cuthbert and Goodheart attempt to infer clinical relevance for MMT diagnosis from studies with positive treatment outcomes. One example cited by the authors in their Table 4 is an observational study by Moncayo et al [32]. The implied logic is that if an AK procedure is used to identify the need for treatment and patients have positive outcomes, then there is evidence that the AK procedure is a valuable diagnostic tool. The flaw in this line of reasoning is that patients can improve despite the diagnostic procedures used. This has actually been demonstrated in a randomized trial evaluating the efficacy of a commonly used chiropractic diagnostic procedure [33]. An efficacious treatment (e.g. spinal manipulation) does not require a valid or efficacious diagnostic test as a treatment indicator [7,33].

### Evidence from randomized trials

The authors note several times in the text that MMT has been investigated in randomized trials. This assertion requires some clarification. In all the randomized trials cited, patients were randomized to treatment or treatment control, and not to diagnostic test or diagnostic test control. This means that the efficacy of treatment was under investigation, rather than the efficacy of the MMT. However, the authors inflated the importance of MMT reliability and validity evaluation by invoking the prestige of the randomized trial; non-randomized cross-sectional/longitudinal studies carry the same weight for the evaluation of diagnostic and prognostic tests.

The efficacy (contribution to patient outcomes) of diagnostic tests and manipulation indicators can and should be evaluated in blinded randomized trials [7,33,34]. We thus agree with the authors' statement that more randomized trials are necessary to validate AK applications of MMT. However, randomized trials of treatment efficacy will not validate AK diagnostics as the authors contend.

Blinded randomized trials can be used not only to evaluate test efficacy, but also to investigate construct validity and the contribution of provocative tests (e.g., joint challenge) to MMT findings. Several construct validity trials of tests used in AK are discussed under construct validity below [23,24].

### Reliability

Reliability is usually considered a necessary but insufficient condition for establishing the usefulness of a diagnostic test [5,6]. That is, poor reliability generally rules out the usefulness of a test (at least in the context of how it is measured [25]), but good reliability does not ensure usefulness. As mentioned above, we do not dispute the reliability of orthopedic/neurological MMT, and are only interested in the reliability of distinctively AK applications of MMT. Several such double-blind studies were omitted from the review [25,35-37].

Jacobs showed good reliability in an unblinded test of sugar solutions but only fair reliability in a double-blind test of MMT response to orally administered oil solutions [35]. Haas et al found poor interexaminer reliability of MMT of a vertebral challenge (muscle "strength" change following directional pressure on the vertebral spinous process) [25]. Two small double-blind studies looked at MMT response to bottled substances held in the patient's hand. Ludtke et al found that response was no better than guessing for both wasp venom and inert substance [36], Garrow showed no test-retest reproducibility of MMT for identifying potential allergens [37]. Pothmann et al. found good intraexaminer, but poor interexaminer (kappa = 0) reliability for muscle tests used for identifying food intolerance in children [38]. Note that we only viewed the English abstract translated from German.

Other reliability studies not included in the review are described below. These were either poorly designed or had negative results.

Peterson found poor reliability in a study of emotional arousal; reliability improved dramatically when confounding variables were taken into consideration [39]. However, this study was poorly designed in that negative confounders were identified and eliminated post hoc using semi-structured interviews, whereas positive confounders were not sought. In Kenney et al, 11 subjects were examined by 3 trained muscle testers for the need of supplementation with 4 different nutrients (zinc, vitamin C, thiamin, and vitamin A) [40]. The examiners did not agree with one another, nor did any of their individual results correlate with laboratory testing, nor was there any correlation of manual and mechanical measures of muscle strength (poor reliability and validity).

Rybeck and Swenson found manual muscle testing (with the Latissimus dorsi), but not mechanical muscle testing, able to discriminate between sugar and no sugar being placed under the tongue [41]. It should be noted that the subjects were not blinded. Although Friedman and Weisberg attempted to test certain AK procedures, their study simply listed the data and lacked any statistical analysis, making it difficult to interpret [42].

### Construct validity

Leboeuf et al investigated the so-called arm-fossa test, a manual muscle testing method used in Sacro-Occipital Technique (SOT) [43]. They evaluated the SOT construct that the arm-fossa test (AK-style muscle test with associated challenge test) is responsive to proper prescribed blocking treatment but unresponsive (unchanged) following improper or no treatment (N = 45). The test returned to normal on follow-up in 73%, 37.5%, and 14% of participants respectively. Results were mixed in this assessor-blind study. In support of the construct, properly treated subjects were more likely to have a normal follow-up than untreated subjects. Contrary to prediction, post hoc testing showed no difference between properly and improperly treated groups, or between improperly and untreated groups (P > .025). Only the properly treated group demonstrated follow-up test results different from mere guessing. It should be pointed out that the evidence is not strong, because of the small sample size and the unblinded subjects.

Important negative evidence was not included in the review: the work of Jacobs et al, Triano, and Haas et al [23,24,35]. Jacobs found that MMT responses to oral solutions were not consistent with AK theoretical expectations in a double-blind experiment [35].

Triano conducted two double-blind experiments (using crossover randomized trial design) to evaluate the AK construct that a weak Latissimus dorsi is associated with the need for pancreatic nutritional supplementation [23]. More specifically, the two theoretical constructs investigated were that a sublingual or cutaneous challenge with pancreatic tissue extract can restore the latissimus dorsi MMT to normal. The control challenges were cardiac, thymic, and testicular extracts that were identified by AK practitioners as unlikely to affect the MMT. There were no differences in post-challenge positive test rates between extracts, indicating no relationship of pancreatic-extract challenge to Latissimus dorsi strength. Triano suggested that future clinical AK research should be informed by constructs developed from basic science studies of AK mechanisms.

Haas et al conducted a double-blind randomized trial, on a mix of participants with and without back pain, to evaluate the relationship of MMT response to a provocative vertebral challenge and to spinal manipulation [24]. They investigated the AK construct that MMT with spinal challenge can be used to monitor response to spinal manipulation. The first phase of the study was a crossover design to compare MMT response of the piriformis to a vertebral challenge and a sham challenge. The second phase of the study was a parallel-groups design to compare MMT response to vertebral challenge in participants either receiving manipulation or receiving no manipulation of the spine. Interestingly, the positive test rates were consistent before treatment across vertebral segments (mean = 5.6%), and post intervention for both treatment and control groups after manipulation at vertebral levels with pretest positive and with pretest negative MMT (8% to 10%). The authors concluded, "For the population under study, muscle response appeared to be a random phenomenon unrelated to manipulable subluxation. In and of itself, muscle testing appears to be of questionable use for spinal screening and post-adjustive evaluation [24]."

There is a recurring theme in these trials. Blinded MMT demonstrates uniform positive test rates, regardless of the presence/absence of or type of the provocative test (e.g., spinal challenge). We can hypothesize that there may be an inherent positive test rate associated with particular muscles. Perhaps this rate is dependent on the patient's state of health. Interestingly, since these positive test rates are fairly small, any follow-up tests, with or without provocative test, have a high probability of being negative. Therefore, clinicians will inevitably think they have successfully treated a condition identified by the original test, despite the fact that the follow-up test results may be independent of intervention. That is, the clinician could be fooled by a statistically random phenomenon associated with a worthless test, a test with results unrelated to provocative procedure and insensitive to spinal manipulation.

### Criterion validity

Cuthbert and Goodheart did not establish the criterion validity for any MMT putatively associated with a condition (neuromusculoskeletal or otherwise) unrelated to a neuromusculoskeletal condition of the same muscle. Thus, they did not present evidence for the criterion validity for any AK challenge or therapy localization test.

The authors do cite a study of a therapy localization test by Pollard et al, which utilized the patient's hand contact on the "ileocecal valve point" in conjunction with a deltoid MMT to identify patients with low back pain (gold standard) [44]. The study showed high sensitivity and specificity of the test. However, the unique effects associated with therapy localization and with MMT of different populations using the deltoid muscle were confounded and the effects of neither component were evaluated. For example, the observed validity could have been due to differing base positive test rates in persons with and without low back pain, and nothing to do with the therapy localization test. The differing positive test rate could be trivially related to distraction or discomfort from the back pain itself, so that the same results could have been obtained from any muscle. Participants were not guaranteed to be naïve with regards to the purpose of the study. These issues could be sorted out using randomized trials as described above. Finally, the high sensitivity and specificity in this particular study are not clinically compelling for two reasons. It does not indicate any specific treatment, and there is a perfectly accurate, cost-effective, and easily performed test available: patient report of low back pain.

The authors did include an early study by Jacobs et al that looked at the correlation of an AK test battery for thyroid function with independent evaluation using clinical signs and symptoms and laboratory tests [45]. Patients were rated on a 7-point scale from unquestionable hypothyroidism to unquestionable hyperthyroidism. The protocol for determining the scale ratings from the battery of test results was not described. The correlation between the AK regimen and other test batteries was r = 0.32 to 0.36, indicating modest accuracy. The results could also be explained by the lack of definitive gold standard or, perhaps, the un-standardized methods of test interpretation.

Missing was Pothmann et al, who found no significant relationship of AK MMT with laboratory tests for identifying nutritional intolerance in children: RAST (radioallergosorbent test) and Cytolisa (sensitivity 73.6%, specificity 45.2%) and lactose breath hydrogen test (sensitivity 77.1%, specificity 43.2%) [38]. The poor positive likelihood ratios (1.34 and 1.36) and poor interexaminer reliability suggest the test performs no better than guessing.

### Reviews and critiques

The authors did not acknowledge previous reviews and critiques of AK. Teuber and Porch-Curren note that several studies refute AK in diagnosis of food allergies and they concluded: "The weight of the evidence to date suggests that this diagnostic modality is not validated when subjected to scrutiny [46]." Tschernitschek and Fink reviewed AK procedures including those used in dentistry. They concluded that there is a lack of evidence for AK effectiveness, reliability, and validity [47]. Haas found that MMT reliability could not be substantiated before 1991 because of methodological and statistical limitations of published studies [9]. Klinkoski and LeBoeuf reviewed scientific papers published by the International College of Applied Kinesiology between 1981 and 1987 [48]. The authors concluded that no conclusions could be drawn because of inadequate methodological quality based on clear identification of sample size, inclusion criteria, blind and naive subjects, reliable test methods, blind assessors, and statistical analysis. Motyka and Yanuck found that the body of AK research is equivocal, sometimes confirmatory of reliability and validity, other times not confirmatory, and often simply irrelevant due to various design flaws [49].

### Diagnosis of preclinical and subclinical disease

AK proponents claim to be able to diagnose preclinical and subclinical conditions [1,16]. Demonstration of the validity of MMT for such conditions would require a comparison to a standard with strong predictive validity of disease, or demonstration that prophylactic care based on AK MMT results prevents or diminishes the development of disease relative to an untreated control group. We could find no such studies.

## Conclusion

Cuthbert and Goodheart conducted a review with important methodological deficiencies. When manual muscle testing as used in Applied Kinesiology is disentangled from standard orthopedic/neurological muscle testing, the few studies evaluating specific AK procedures either refute or cannot support the validity of AK procedures as diagnostic tests. In particular, the use of MMT for the diagnosis of organic disease or putative pre/subclinical conditions is insupportable.

## Competing interests

The author(s) declare that they have no competing interests.

## Authors' contributions

All authors critically appraised the Cuthbert and Goodheart review. Haas wrote the initial draft. Cooperstein and Peterson added material to subsequent drafts. All authors reviewed and approved the final submission.
